# Practitioner Perspectives on Delivering Integrative Medicine in a Large, Acute Care Hospital

**DOI:** 10.1155/2015/394040

**Published:** 2015-11-26

**Authors:** Kent C. Nate, Kristen H. Griffin, Jon B. Christianson, Jeffery A. Dusek

**Affiliations:** ^1^Integrative Health Research Center, Penny George Institute for Health and Healing, Allina Health, 800 East 28th Street, MR 33540, Minneapolis, MN 55407-3799, USA; ^2^Division of Health Policy and Management, University of Minnesota School of Public Health, 420 Delaware Street SE, MMC 729, Minneapolis, MN 55455, USA

## Abstract

*Background.* We describe the process and challenges of delivering integrative medicine (IM) at a large, acute care hospital, from the perspectives of IM practitioners. To date, minimal literature that addresses the delivery of IM care in an inpatient setting from this perspective exists.* Methods.* Fifteen IM practitioners were interviewed about their experience delivering IM services at Abbott Northwestern Hospital (ANW), a 630-bed tertiary care hospital. Themes were drawn from codes developed through analysis of the data.* Results.* Analysis of interview transcripts highlighted challenges of ensuring efficient use of IM practitioner resources across a large hospital, the IM practitioner role in affecting patient experiences, and the ways practitioners navigated differences in IM and conventional medicine cultures in an inpatient setting.* Conclusions.* IM practitioners favorably viewed their role in patient care, but this work existed within the context of challenges related to balancing supply and demand for services and to integrating an IM program into the established culture of a large hospital. Hospitals planning IM programs should carefully assess the supply and demand dynamics of offering IM in a hospital, advocate for the unique IM practitioner role in patient care, and actively support integration of conventional and complementary approaches.

## 1. Introduction

In this paper, we describe the delivery of integrative medicine (IM) at a large, acute care hospital from the perspectives of IM practitioners. IM refers to the integration of conventional and complementary medical approaches (e.g., acupuncture; massage; mind/body interventions) to provide a model of care drawn from both, as needed and appropriate for patients. Programs providing IM to inpatients are relatively new, and many have been targeted for a specific population (e.g., pediatrics [1–3], oncology [4–6]).

Previous research on IM in hospital settings described the development and operational elements of inpatient IM programs [7–10] or focused on nursing, administrative, or physician perspectives [11, 12]. For instance, the authors of one study highlighted the views and experiences of administrators regarding the development and ultimate failure of an IM center [7]. Other studies have obtained IM practitioner perspectives in the context of primary care and research settings [13–17]. One qualitative study reported the findings of interviews with complementary medicine practitioners working in hospitals in Israel, but these practitioners delivered care to ambulatory patients or outpatients on the hospital campus, rather than to inpatients at the bedside [18]. Another study interviewed five acupuncture fellows working in a short-term, exploratory program with inpatients [19]. The literature to date has not addressed the delivery of IM care in an inpatient setting from the perspective of IM practitioners employed by the hospital.

## 2. Materials and Methods

### 2.1. Study Setting

Abbott Northwestern Hospital (ANW), a 630-bed tertiary care hospital, has had an inpatient IM program in place since 2003. Its development was described previously, as were patient outcomes after receipt of IM through the program [20–23]. IM services are available at no charge to all hospitalized patients from 9 am to 5 pm, Monday through Friday. The program is funded by ANW with additional support from the Abbott Northwestern Hospital Foundation and the Penny George Institute Foundation. The program has shifted from its initial model of placing IM practitioners in specific hospital units to, currently, sending practitioners across the hospital in response to electronic health record- (EHR-) based referrals placed by physicians, nurses, and other providers.

In 2014-2015, the IM team consisted of 16 practitioners, with specialties including acupuncture (*n* = 6), massage therapy (*n* = 8), holistic nursing (*n* = 1), and music therapy (*n* = 1). All practitioners were credentialed in their areas of expertise. These practitioners were hired based on their experience delivering their respective integrative medicine modalities, and several, who had nursing backgrounds, had experience working with hospitalized patients. In addition, their training at ANW included education on the delivery of modalities such as relaxation techniques and aromatherapy. They were trained in providing care in the hospital setting by shadowing other practitioners before practicing alone with patients. At the time of the study, all practitioners on the team had been employed as IM practitioners at ANW for over one year, with the majority having been part of the program for between eight and ten years.

Referrals to IM were made by clinicians (primarily nurses) using specific referral processes within the EHR. Physicians could refer for acupuncture or general IM consults, while nurses and other clinicians (e.g., physical therapists) could refer only for general IM consults. After receiving referrals through the EHR, IM practitioners used a numerical scoring system as well as their clinical judgment to triage patients. The triage process was conducted each morning before practitioners began visiting and treating patients. Because this process was a topic of substantial interest and discussion in our interviews with practitioners, we describe the steps in more detail in Section 3.1.1 of Results. Practitioners provided treatment to patients who were determined suitable to visit, after consulting with the patient and obtaining the patient's permission to be treated. In addition to providing therapeutic IM treatment, practitioners also educated patients and family members on self-care techniques for coping and symptom management.

After treating patients, the IM practitioners entered data pertaining to each IM session into a medical progress note as well as a customized EHR documentation flow-sheet, where they recorded any information specific to the visit and modality administered (e.g., acupuncturists listed points and meridians used). In addition to documenting basic information like treatment start and end times, practitioners asked patients to rate four items from 0 to 10: their pain, anxiety, nausea, and current ability to cope. These metrics were tracked by the hospital for operational/quality improvement purposes. During the interview period, the 0–10 scores collected by practitioners on pain and anxiety were also being used for a research study.

During 2014, there were 5,193 referrals placed for general IM consults at ANW. Patients were not approached by IM practitioners in 530 cases, typically because of the lack of IM practitioner time, or discharge of patients before the patient could be approached for services. Of the 4,663 who were approached, 3,244 were seen by a general IM practitioner, while 1,419 were seen by an acupuncturist. As a consequence of the general IM practitioner visits, 2,106 patients received IM treatment. The primary reasons that IM services were not delivered after initial contact were that the patient was unavailable (*n* = 490) or judged not to be in need of services after IM practitioner assessment (*n* = 299). Out of the 1,419 visits by acupuncturists (resulting from a general IM referral), 434 patients received treatment.

During the same period, there also were 559 referrals made specifically for acupuncture. This resulted in 448 patients being approached either by an acupuncturist or by another IM practitioner (e.g., massage therapist). Of these patients, 256 ultimately received services from an acupuncturist, while 53 received (nonacupuncture) services from another IM practitioner.

### 2.2. Participants

We sought to interview IM practitioners who were practicing with the IM team at the time of data collection (spring of 2014). Interviews were also conducted with holistic nurses who had previously been part of the team but had recently moved to an adjacent (learning and development) department. Our goal was to recruit 15 practitioners in total. Invitations were emailed to prospective participants from the study PI (Jeffery A. Dusek) and follow-up contacts were made by the study coordinator (Kristen H. Griffin) if invitees did not respond to the initial invitation. Ultimately, in-depth interviews were conducted with 15 IM practitioners, including four licensed acupuncturists, seven certified massage therapists, one certified music therapist, and three certified holistic nurses.

### 2.3. Data Collection and Analysis

A structured interview protocol was approved by the institutional review board, with questions for practitioners pertaining to their background and training in their modalities, the process of triaging IM referrals, the process of delivering IM services, challenges in delivering IM in a hospital setting, and overall views on the hospital's IM program, including their suggestions for improvement.

Of the 15 IM practitioners who completed interviews, two were former and 13 were current members of the team. Three current practitioners declined to participate citing lack of interest, and one former member of the practitioner team did not respond to the invitation and follow-up correspondence.

Interviews were conducted between January 27, 2014, and April 8, 2014, by Kristen H. Griffin and Jon B. Christianson. All interviews took place in a private room at the study team's office, and interviews were recorded after the participants' consent to participate and be recorded was obtained. Interviews ranged from 30 to 77 minutes long. Interviewees were assured that any comments they made would not be attributed specifically to them.

Recorded interviews were transcribed by an independent transcriptionist, and ATLAS.ti software (version 7.5.4) was used to organize and code the transcripts. A basic coding structure was applied to each transcript, using the main topic areas in the protocol questions. Two researchers (Kent C. Nate and Kristen H. Griffin) then used grounded theory principles [24, 25] to complete a second round of coding. The study team met regularly during coding to discuss emerging themes. Data saturation was reached [26]. Additional details regarding interview coding and analytic approach are contained in the Appendix (see Supplementary Material available online at http://dx.doi.org/10.1155/2015/394040).

## 3. Results

Based on the interview data, we identified three major themes regarding practitioner perspectives on delivering IM in a tertiary hospital: ensuring efficient use of IM practitioner resources, defining the IM practitioner role in affecting patient experiences, and navigating the differences in IM and conventional medicine cultures in an inpatient setting.

### 3.1. Efficient Use of IM Practitioner Resources

Demand for IM services often outpaced the availability of practitioners to deliver requested services, forcing them to prioritize patients. Practitioners discussed several issues associated with ensuring proper allocation of resources: use of an EHR rating system, the nuanced nature of provider preferences in the triage process, the appropriate timeline for service delivery, and, to some extent, the availability of patients.

#### 3.1.1. Use of an EHR Rating System for Patient Triage

IM practitioners exhibited varying degrees of support for the use of an EHR “workbench” to rate and prioritize incoming referrals. When a provider in the hospital entered a referral for IM into the EHR, along with a note describing the reason for the referral, information about the referral and the patient was compiled into a report for the practitioners to review as a team before the start of each workday. This workbench used a spreadsheet interface to display information including each patient's name, location, referring provider, and reasons cited for referral, including notes about symptoms such as pain and anxiety over the past eight hours. The report used symptom-based ratings to place patients into a priority order for being seen by IM providers. Practitioners then met in a large group (rounds) to discuss each patient and decide which patients actually would be seen and which practitioner would visit the patient. New and returning patients were included in the report.

The use of the workbench and priority rating was introduced in July of 2013. When the IM program began, practitioners received phone calls from referring providers and tracked requests manually. This process was replaced after the hospital implemented an EHR by a system of paper orders originating from the EHR. The workbench was developed as a way to triage patients more objectively, using an automated system.

Some IM practitioners saw the workbench system as playing an important positive role, while others felt that it inhibited practitioners' ability to help the “right” patients. For the latter practitioners, the systematic approach to rate patients was perceived, at least sometimes, to be at odds with the important principle of trusting one's intuition about what patients should be seen.

*That numbering system for me is just** wrong**…the nurses are rating pain but every time they go in the room, they have to rate [the patient's] pain. Part of me gets it, but pain, my understanding is pain management. They [could] say “how is your pain, is it high, medium, low?” That's OK in my world, I could answer that, but to** have** to think of a number, it just seems wrong to me. (Emphasis speaker's)*

But, another practitioner observed is as follows.

*It's really important because you want to make sure we're seeing the best patients that we can see…the people who have the most need for it…I know some people didn't really like [the workbench] at the beginning because it was too different from what they were used to. I was used to being shuffled, you know, “I'll do whatever, you want me to see that patient, that's fine with me, I don't care.” I didn't have a set thing in my mind that I had to do every day. *

Whether practitioners were more or less comfortable using the workbench, no one described it in black and white terms, instead acknowledging its potential value or challenges (e.g., in the quotations above: “part of me gets it, but…”; “I know…it was too different from what they were used to”).

#### 3.1.2. Provider Preferences

Many IM practitioners stressed how nuanced the prioritization process could be. Often, there was interplay between the rating system and practitioner preferences in determining how the limited IM resources would be allocated. For example, inpatient units where physicians and nurses had strong existing relationships with practitioners tended to receive higher prioritization from practitioners. Typically, the referrals generated from these clinicians were described as “higher quality” referrals by the IM practitioners.

*Another thing is also the quality of the referral…Two of the flags that are available for patients to be prioritized are the referring provider and the unit. So there are certain providers that we know…work really well with us, and they customarily refer and give good referrals. *

Additional factors that influenced the triage process included relationship with the patient; if the patient had been seen before; a patient's actual or expected length of stay; and recognition of the subjectivity inherent in asking patients to report pain scores with a number between 0 (no pain) and 10 (highest pain).

*It's at rounds where we divvy patients out and it's usually based off of our previous relationship with them…it's more nuanced…because numbers, though they are concrete and objective…people can make any meaning they want out of them, you know, and they're also very subjective at the same point. You know there could be a five and for some people that would be really high, and some people, it would be really low. And people's threshold for pain is really different, and so the way they, they call out for help is also really different…but if we have a relationship you can kind of assess the nuances of how someone describes pain. *

According to a different practitioner, a patient's length of stay in the hospital can also factor into the patient-practitioner dynamic.

*And then on the other side of that, sometimes you have a relationship with someone and because you know them, and they'll be here for a long period of time, you can maybe skip them that day because you know you'll try to see them in the next day or so. And then there's people who are here just for a few days and you want to get to see them, so it's a constant weighing out of things. *

#### 3.1.3. Timeline for Service Delivery

The process by which practitioners were allocated also was affected by the timeline of the delivery of services. Upon initial assignment, IM practitioners sought to see patients within twenty-four hours. Typically, visits did not occur until the day after the referral was initiated. For referrals placed on Fridays, this meant patients were not seen until the following Monday, as IM services were only available Monday through Friday. Furthermore, referrals reviewed in morning rounds, but not assigned due to limited resource availability, were entered into a queue on the EHR workbench. As practitioners completed visits, they pulled additional patients from this queue.

*The other piece of the process is that we still do look at people that were not picked up the day previously because of lack of resources. We may go through that list to see if because they were skipped, you know, we'll reassess, ok where are they, how many flags do they have, if they still haven't been seen, do we need to prioritize them? *

#### 3.1.4. Patient Availability

About half of the practitioners mentioned the inability to assess patients after receiving an assigned referral because the patients were not available in their hospital rooms. This may have been because patients were sleeping, were in consultation with another provider, or were receiving tests/services elsewhere in the hospital. There was a loss of IM practitioner productive time when patients were unavailable. Practitioners specifically mentioned the challenge of limited resources with regard to their ability to provide services to patients in need. Some practitioners struggled with the frustration this created.

*[The limited resource availability queue] is another way of saying, “If I could, I would see them, but I don't have time.” And so sometimes now, patients in that category are noted on the workbench as being in that category, so that they would be the priority for people to see if they ended up having time. Or they would be the priority for the next day because they would have been seen today, but there weren't resources. And that whole area of people not being seen is an area of tension to me. It's been frustrating to me sometimes that it feels like, it's easy to feel like there's scarcity, there's not enough, and it's a hard way to start the day. *

Meanwhile, other practitioners described ways that they—as individuals and as a team—coped with the reality of being unable to see every patient referred for services.

*We do this blessing every morning and it's about whoever is referred is the right person, whatever work comes is the right work and whenever it happens is the right time, and I mean, for me to function, I really have to put my trust in that because there's days where we just have lots of people who aren't going to be seen. *

### 3.2. Defining the IM Practitioner Role in Affecting Patent Experiences

After it was determined which patients the practitioners would visit, much of the IM practitioners' day-to-day work process revolved around interactions with patients and family members. Practitioners emphasized several elements related to how they worked to optimize the patient experience: empowering patients to have a choice in accepting or declining IM services; shared decision-making with patients regarding what kind of treatment to deliver; and a range of common interactions with patients' family members.

#### 3.2.1. Patient Empowerment

All of the IM practitioners were comfortable with patients declining their services. It was common for patients to be concerned with the cost and effectiveness of IM treatment. Also, some patients declined services outright due to religious beliefs. If practitioners were unable to mitigate these concerns by assuring patients that there was no cost or by providing education, they supported the importance of patients being able to decline services. When describing these experiences, practitioners used very similar language in affirming the patient's ability to decline services. Practitioners saw this as a crucial opportunity to empower the patient in a situation (being hospitalized) in which patients tended to feel they had little input into the care they received.

*The piece too that I tell people, I tell nurses this all the time, and I tell patients, there is** great empowerment** to say no to me. You cannot say no to anybody else. You got to say no to me. That** is** a plus thing. Or for me to come and offer a session and for them to go, “You know what, I'm really feeling pretty good today.” Oh, my god! The nurses will go, “Are you kidding me? You've gotta let her do something. She's amazing!” And I go, “Wait a minute, wait a minute, no, no, no!” There's** empowerment** in saying no to me. (Emphasis speaker's)*

#### 3.2.2. Shared Decision-Making

When patients decided to receive treatment, practitioners developed treatment plans jointly with patients, viewing their own role as that of a partner in providing care. They also sought to thoroughly understand the patient's physical symptoms and causes. Practitioners described this process as assessing the patient holistically. Relying on personal experience and clinical intuition, they provided a series of treatment options they felt would be most helpful and would allow the patient to make the final treatment decision.

*Well, I'd kind of introduce services to them and let them start to explore what they might find helpful…You know, everybody I was working with was dealing with pain, anxiety, nausea, constipation, so we'd kind of come up with a little laundry list of “well this can help with this or this can help with that,” and sometimes you would just rely on the intuitive hit, where, you know, I'd walk in with somebody and I'd just kind of get this, “in the forehead” sort of experience where I haven't used this technique with somebody in a long time, but I think this is the right person to use it for, and those usually turned out to be fairly accurate, so I've learned to trust them.*

#### 3.2.3. Relating to Family Members

Additionally, the experience of the patient in accepting services and/or specific IM modalities sometimes was influenced by the presence of family members. When family members were present, IM practitioners reported that they sought to incorporate them into the care discussion in a welcoming and cooperative manner. Family members varied widely in their views of IM, with some being highly supportive, others less supportive, and some overtly resistant to the delivery of care. Irrespective of family perspectives, practitioners attempted to ensure that the decision for treatment was ultimately made by the patient.

*If they have family in the room, then that's a whole other thing because then there's a distraction. I usually try to include them. You know it depends on the feel of the room. I always go in by the feel, you know. So if they're smiling and laughing, and you know, interacting with the patient, then I just kind of reflect what they're doing. If they're sitting there staring and nervous, I tone everything down. I just mirror what they're doing in the room. In order to be respectful. *

Practitioners not only included family members in the decisions and discussions surrounding IM treatment, but they sometimes taught them simple techniques (e.g., breathing, relaxation approaches) that they could use with their hospitalized family member after the IM session, or even the hospitalization, had ended. Practitioners felt that the role of education with both patients and family members was an important component of their work.

### 3.3. Navigating the Differences between IM and Conventional Medicine Cultures

During the interviews with IM practitioners, cultural issues associated with providing IM services in an inpatient setting emerged. Practitioners described the following elements related to this theme: the complicated nature of practicing within a large medical center in general; the challenge of integrating an unfamiliar service in that type of environment; and the overall difficulty, but also gradual progress, associated with building a business case for this service.

#### 3.3.1. Complicated Nature of a Large Medical Center

Many of the IM practitioners employed at ANW previously had worked in outpatient settings. As a result, their transition to providing services in a large inpatient setting often was challenging. In particular, some practitioners mentioned the initial difficulty of acclimating to the complex physical layout of the hospital.

*I think the thing that requires additional support, you know, for one, it's a large hospital, and so finding your way around to be efficient was something that took some time. But I think that's such an individual thing, and now looking back on it, I think there might be more efficient ways to sort of learn how the system, how the hospital's laid out so that you can learn how to be efficient at making your way around the hospital. And I guess it's not so much from a delivering therapies standpoint, as much as just how to work within the Western setting. *

For some practitioners, the initial challenge of learning how to navigate the large hospital setting most effectively was followed by planning for the hospital-wide workload that could be required on any given day. This daily planning involved accounting for time spent walking to and from various parts of the hospital, a process that could be frustrated by patient availability. On a related note, practitioners described a hospital-wide culture focused on productivity and operational efficiency. Such a culture often stood in stark contrast to practitioners' previous experience in other work settings. According to one practitioner,

*We have a very big concern about wasting time. There's this process improvement work that's been happening in our department, and there's an increasing, I'd say, you know, compared to other departments, I think that it's been pretty relaxed for us, but there is sort of a very clear message that we should be increasing the amount of time that we spend with patients…We're asked to, you know, be more productive. *

Another said the following.

*I think like all of the other departments, we are coming under more scrutiny, more time crunches. When we started, we had the luxury of providing the care that we felt was needed and appropriate, for the most part independently, and I think now there's a lot more focus on, well you've got this amount of time to do this amount of services. And we're trying to make it the most efficient system and you know that for any practitioner of any services that's going to be a little more limiting in the care they can provide. *

#### 3.3.2. Integrating IM Services into an Inpatient Setting

Many practitioners noted the importance of helping clinicians understand how IM services are complementary, not alternatives, to conventional medical treatment. To some extent, the culture of the organization had evolved to accept this perspective. For example:

*My orientation to start working on the inpatient side revolved around the idea that the services that our department offers are not an alternative to, but an adjunct, you know, part of the care that patients then receive. So it's not an alternative medicine, it's integrative, in the true sense of the word. So we bring the best of what our modalities have to offer, and add that to all the good work that the Western medical model is delivering. The orientation that I had was basically around figuring out how to adjust the work that I do as a massage therapist to the different health populations that we work with. And then, also learning about the other modalities that our department offers so that I can more effectively refer patients around, you know, based on what they need. *

However, some practitioners felt that true “integration” had not been achieved, but was still very much “in process.”

*It's a really nuanced thing, and what I see happening all over is people throwing different practitioners together, but to me, that's not integrative medicine, it's really different…it's not just kind of throwing people together. We're not integrative medicine really. We are** integrating** medicines but we haven't reached that yet, and it's a process. So, I'm glad that that's our name and that's what people call us, but I think there's a lot to becoming integrative medicine. (Emphasis speaker's)*

Interestingly, some practitioners mentioned the quality of referrals they received as an indicator of the overall cultural awareness and acceptance taking place within the hospital.

*We always try and check in honestly with the patient and really assess their needs, but typically there are certain patients, certain referrers who kind of don't get it. They don't understand what it is that we do. They think that, you know, “oh, you know while you're here you can get massage.” So then, they request us, and we come, and the patient is just like, “Oh yeah, everybody said that I should get the foot massage, ‘cause they said it's awesome.” And, that is really missing the mark. And so anytime we get a hint of that in the referral, that there's kind of like a, you know, “Oh I just want it, Oh I just thought it would be nice,” then our team as whole I would say really moves away from seeing those people as a priority. *

#### 3.3.3. Building the Business Case for IM Services

Historically, the ANW IM inpatient program developed somewhat organically, with a few physician and nurse champions supporting its hospital-wide dissemination. However, recognizing the need for wider acceptance and support, operational efficiency efforts and research investigations were undertaken. Reactions from IM practitioners themselves to these efforts varied widely; some believed they were necessary for the wider acceptance of the program by conventional clinicians, while others believed they undermined the original purposes of the program. In particular, some practitioners felt that quantifying pain, anxiety, and other patient-reported outcomes (either for research or operational purposes) oversimplified the services they offered to patients in terms of a more holistic approach to healing.

*We were pretty much the first hospital in the country to do this in the way that we're doing it, and I think the unique beauty of the way the program developed was that we were under the radar and we weren't considered a threat, and we had a nurse who was well-respected and completely unthreatening because she was a nurse, dispersing this team throughout the hospital, and we had a group of physician champions, who said “yes, go.” Over time, that's evolved and now we're above the radar, and being looked at, and I would say the evolution is the challenge. The research aspect from my perspective is a challenge. It's a dialogue we've all longed to be part of, and until recently, you know our role has been minimal…so I think the greatest challenge we have is** how** to identify the right questions to be asking. I think pain and anxiety are not reflective of what we do and so the research part is a unique challenge to what we do, and that's new in the scope of things, that's relatively new to the process. The story I always tell people is that when I first arrived here, we would put our sign on the door and be halfway through a session, the lights would be dimmed and a physician would barge in with a cold stethoscope and turn the lights on and say “I'm rounding,” and about six months into it, there'd be a gentle knock on the door, and the physician would say, “I'm really sorry, I'm rounding,” and they wouldn't turn the lights on and they'd warm the stethoscope and then after about a year, I came out of a room and a cardiologist was sitting on a bench and said “what were you doing in there?” and I told him, and said “the patient's sleeping, but I'm sure he'd like to see you” and he said, “no, if he's sleeping, I'll come back later,” so there's been this evolution of regard and respect for what we provide over time. *

Practitioners said they took pride in and enjoyed their work and felt supported by their team of fellow IM practitioner colleagues. Yet, the work (and the program in general) was described by many as a balancing act between growing acceptance on the part of patients and other clinicians and an intensifying sense of being under pressure to quantify their outcomes.

## 4. Discussion

### 4.1. Limitations

Our findings from this qualitative study may not be generalizable to all hospitals. The IM program at ANW is well-established at a single facility and has features that some hospitals may find difficult to duplicate (e.g., number of IM practitioners, structural process for prioritizing referrals). In addition to the program being well-established, most of the practitioner team had worked for many years within ANW, so their experiences may not be applicable to newer programs. For example, because many of the practitioners interviewed in this study had been with the ANW IM program since its earliest days, these practitioners' hesitancy to use numerical-based systems for triage and assessment may be linked to having been with the program before it relied on such measures. However, the hospital environment at ANW is not necessarily unique among hospitals of similar size and scope in the US. Therefore, other hospital-based programs at various stages of development are likely to benefit from considering the perspectives of IM practitioners reported here.

Beyond study limitations, the ANW IM service may have programmatic features that limit optimization. When we asked interview participants what changes they would suggest for the delivery of IM at ANW, most of the responses centered around more satisfactorily finding and treating those patients who could most benefit from IM. In other words, the practitioners see room for improvement in how the “right” patients are referred. Responses included wishes to see a different triaging system besides the workbench, higher staff levels to meet demand, and generally more trust and integration of IM practitioners by the dominant western medical culture and providers at ANW. Some additional challenges, including the subsidized nature of the program and the onboarding process for practitioners, are addressed in Practice Implications.

### 4.2. Practice Implications

Our research highlights at least three major implications important to the practice and assimilation of IM into inpatient settings. First, there is likely to be a delicate balance of supply and demand of IM services in any large acute care setting. Second, IM practitioners have unique potential to provide holistic and empowering care to patients. And third, there is a need to effectively orient and train IM practitioners in the cultural dynamics of a large hospital.

ANW's IM program, presently a free service for patients, is delivered to a large hospital population by a relatively small team of practitioners. In the context of these limited practitioner resources and the large patient load, the program focuses on how to appropriately allocate practitioners to patients who are determined to be the most suitable or in the most need. In interviews, practitioners frequently cited the limitations of a prioritization system based on pain and other symptom scores, suggesting that many factors could affect the process of triaging referrals and providing services to patients. A systematic approach seems, at best, to be a helpful starting point for some practitioners in managing high demand. At ANW, patients' ability to receive these services at no charge was seen as a valuable and unique opportunity for them to experience the benefits of integrative therapies. But the reality is that the structure at times stretched IM practitioners thin and introduced unique challenges and frustrations into their practice. Funding has been a challenge for some other inpatient IM programs [7, 8], and a review of integrative oncology programs (outpatient and inpatient) reported that most of these programs provided at least some IM therapies free of charge to patients, with funding and cost recovery mechanisms including combinations of foundation and grant support, hospital budgets, third party billing (outpatient only), and billing to patients [4].

Due to the increased prevalence of a productivity-driven culture, it is possible that nurses may feel they are not able to provide some of the services directed at promoting comfort and relaxation that traditionally were part of nursing practice, (e.g., offering back massage to patients). A powerful role for IM practitioners in a hospital setting is in supplying some of the less tangible elements of a healing environment: time, listening, and empowering patients in shared decision-making about treatments. Integration of IM programs may occur more easily through efforts that communicate to conventional medical staff this important role to be played by IM practitioners.

It was clear from the interviews that the shift from the original mode of delivering inpatient services (less systematic, more intuition based) to the newer, “scrutinized” delivery method was jarring for many practitioners. Participants expressed hesitations about quantifying patient outcomes and basing triage on standardized measures. These types of concerns underscore the challenge of integrating a conventional and a complementary treatment paradigm.

Besides shadowing other IM practitioners, most practitioners did not describe receiving any training that would have better prepared them for entering into the inpatient environment of a large medical center. Practitioners with hospital nursing backgrounds tended to experience an easier transition to provide IM in the inpatient setting. However, in recent years, the onboarding process for the ANW program has become more structured. Other existing or prospective hospital-based IM programs should prepare for a practitioner learning curve related to simply working in the inpatient setting, particularly if practitioners' backgrounds are in outpatient or community settings.

It is worth noting that acceptance by conventional providers and administrators takes time and is an evolving process for all parties involved. Integrative medicine, despite a growing presence across the US healthcare landscape, is far from universally accepted or embraced by conventional medical clinicians and decision-makers. Physicians have expressed resistance to their patients' use of complementary or alternative therapies [27, 28], and communication between physicians and patients about use of these therapies is moderate at best [29–34]. However, this resistance is generally related to patient use of alternative or complementary therapies when these therapies are not integrated into the conventional course of care. There is some recent evidence of support for the integration of medical models. For example, in a recent qualitative study with physicians, nurses, and administrators at a large veterans' medical center, participants praised the potential of IM to provide more patient-centered care than conventional medicine alone [35]. Still, hospitals where conventional and complementary medicine providers work together to treat patients are relatively new terrain. Those in the positions of authority at hospitals and health systems may not be convinced across the board of the value of IM. Therefore, as some of our interview participants mentioned when describing the nurse-led creation of the ANW IM program or the “good” referrals of certain physicians, it will be important for new programs to use relationships with conventional practitioners who can serve as champions. IM practitioners also described a growing acceptance and acknowledgement of their presence and services over the course of the program. Because our interview sample was comprised primarily of practitioners who had been with the program for all or most of its existence, they were well qualified to reflect on the changes in attitudes and culture surrounding integration at ANW.

More extensive training, that is specifically focused on a hospital's culture, may be helpful for IM practitioners, just as consistent and targeted training of other clinicians in how best to work with IM would help to facilitate integration. Furthermore, collection of data for research and evaluation may bolster administrative support for integration. Some previous research has noted the need to better prepare all players for the adjustments and challenges of integration. For example, there is evidence of the effectiveness of training complementary medicine practitioners, while they are students, in integrating with conventional providers and practices, suggesting the value of preparing IM practitioners for a setting like an IM inpatient program even farther upstream than when they begin training for a role at a hospital [36]. And an expert panel on creating an integrative medicine service or department recommended “overcommunicating” the aims and expectations of integration to various stakeholders (including complementary practitioners and the conventional providers with whom they would interact) [37].

Although no other studies have examined a program analogous to ANW's IM service, some of our findings are consistent with those of other research regarding complementary medicine practitioners in various settings. One study used interviews to examine the experiences of acupuncturist fellows working with inpatients in an exploratory program at a hospital [19]. Although there were some key differences from our participants (e.g., the acupuncturists were not paid employees of the hospital and had less experience in the inpatient setting than our sample), the acupuncturists in that study described some similar experiences and perceptions. For example, the faster pace of the hospital setting (versus private practice) forced practitioners to compromise a broader holistic model in order to treat patients in a more targeted, time-efficient manner. They also expressed pride and enjoyment in their unique role in improving patient experiences. A qualitative interview study on the topic of patient expectations included IM practitioners' views that patients must play an active role in their healing [17], a theme consistent with the focus on patient empowerment and shared decision-making in our study. Another interview-based study with complementary medicine practitioners raised the topic of how complementary therapies provide a paradigm of healing that has largely been impinged upon by the pace and demands of conventional medicine [38]. Practitioners in this and another study also discussed their roles in empowering patients to facilitate their own healing [39]. Complementary medicine practitioners in other settings appeared to face similar challenges and to identify similar strengths in their role as did practitioners in ANW's inpatient program.

### 4.3. Future Directions

Future research on hospital-based IM programs should examine patient perspectives on the presence and/or receipt of IM in hospital settings. One study of a hospital IM initiative reported survey and qualitative interview results from patient participants [5], although the patient population was confined to a small sample of oncology patients. However, patients in that study provided substantive feedback regarding aspects of the program they found more or less appealing and helpful in their care. More such research in a broader inpatient sample would provide valuable insight into optimizing patient experiences, particularly if coupled with patient satisfaction measures.

## 5. Conclusions

This study describes the experience of IM practitioners delivering therapies to inpatients in a large hospital and explores the primary features and challenges of such a program from the standpoint of these IM care providers. With over a decade of experience, the IM program at ANW is well-established and generally works well for those practitioners delivering its services, although challenges exist in balancing supply and demand and in navigating the culture of a large hospital. IM practitioners perceive the uniqueness of their role in patient care at ANW, a role that includes providing an opportunity for truly shared decision-making, as well as education on self-care techniques that patients and their family members can take beyond the hospital. The team of IM practitioners could benefit from further integration and better awareness by other health care providers of the role for IM in patient care. Other hospitals with IM programs planned or in progress can learn from the challenges and strengths of ANW's IM inpatient service.

## Supplementary Material

The supplementary describes the methods for our study of integrative medicine practi-tioner perspectives on delivering care in a large hospital. This document provides greater detail than was appropriate for the manuscript on recruitment, interviewing, data management, and analysis.
