# Supplementary material for: Practitioner Perspectives on Delivering Integrative Medicine in a Large, Acute Care Hospital
Source: Evid Based Complement Alternat Med. 2015 Nov 26;2015:394040. doi: 10.1155/2015/394040 (PMC4674616; doi:10.1155/2015/394040)
Supplement: Supplementary file 1 — The supplementary describes the methods for our study of integrative medicine practi-tioner perspectives on delivering care in a large hospital. This document provides greater detail than was appropriate for the manuscript on recruitment, interviewing, data management, and analysis. [file 394040.f1.pdf]

Practitioner Perspectives on Delivering Integrative Medicine in a Large, Acute Care Hospital: Methods Appendix

Selection of Interview Participants

Our goal was to recruit 15 integrative medicine (IM) practitioners at Abbott Northwestern Hospital (ANW) who were actively delivering or who had delivered services to inpatients in the Cardiovascular, Mother Baby, Neuroscience and Spine, Orthopedics, and Oncology clinical service lines during a portion of quantitative data collection from a hospital-wide study of pain and anxiety among inpatients receiving IM. The IM service is housed in the Penny George Institute for Health and Healing (PGIHH) at ANW.

All IM practitioners were identified from the Allina Health employee information network or staff rosters provided by department management. The study population consisted of 16 practitioners who were actively providing patient care, and three practitioners who previously had been part of the team during the study period.

Recruitment and Scheduling

The study PI (JD) initially proposed the study to all potential participants via an emailed letter. A "Common Questions" document and a consent information document accompanied the email letter and provided additional details on the study. Following low initial response rates, a follow-up contact was made by JD and KG attending a staff meeting of the IM practitioner team to describe the study briefly and answer any questions that people may have.

If a potential participant was interested in learning more about the study and/or scheduling an interview, they were asked to contact KG. KG scheduled all interviews and tracked recruitment status in a private Excel spreadsheet. Those who declined to participate communicated to KG that they were not interested (no reason given) or were too busy. Four invited individuals (three active practitioners and one former practitioner) declined to participate. Decisions to participate were communicated either to JD or KG via email or telephone. A total of 15 individuals did participate. This group included 13 current practitioners and 2 nurse clinicians who had worked in direct patient care with inpatients during a portion of the larger study but had since moved on to other roles with PGIHH.

Recruitment began on January 13, 2014 and the final interview was scheduled on March 17, 2014.

Interviews and Consent Process

Interviewers

KG began the study as a Research Coordinator and her title changed to Associate Scientific Advisor during the course of the study. KG was trained in qualitative research methods and analysis and participated in qualitative data collection, management, and analysis on a range of projects for several years before this study. JC is an experienced qualitative researcher, who as a consultant with the PGIHH Integrative Health Research Center,

designed the protocol and interview questions for this study. Before data collection, both interviewers participated in a practice interview session to establish similar approaches to using the interview protocol with prospective participants.

KG had minimal to no interaction with the participants she interviewed before the study. JC had no interaction with participants before interviewing them.

Interviews

KG conducted 14 interviews, and JC conducted one interview. Interviews were conducted between January 27, 2014 and April 8, 2014, in a private room at the research team's office, during the practitioners' workday.

An interview protocol was created by the research team and approved by the IRB. Questions went through several rounds of revisions by the team, and were tested in practice interview sessions among the interviewers. As data collection was underway, some commonly-used prompts were added after receiving approval from the IRB. IM practitioners were asked about background and training in their modalities, the process of triaging IM orders, the process of delivering IM services, and barriers/challenges in delivering IM in a hospital setting.

Interviews ranged from 30 to 77 minutes long. Some interviews went longer than scheduled, but were continued until all questions had been asked, after the participant gave permission for the interview to continue. Some participants talked with KG about her credentials and experience, and for assurance of confidentiality. Participants were aware that interviewers were involved with the research department, which they knew to be responsible for quantitative data collection, even though the individual interviewers were not involved in collecting data.

Each participant was interviewed only once. All interviews were recorded on a handheld Olympus DM-620 digital voice recorder and immediately following the interview the digital files were saved to a private folder on the server only accessible to interviewers, and erased from the recording device. Notes were made by the interviewer during and/or after each interview. Immediately following each interview, these notes were scanned and saved to a private folder on the server only accessible to interviewers, and hard copies were securely destroyed.

Consent Process

The verbal informed consent process took place at the beginning of each interview, before recording was begun. Participants were asked if they had read and understood the consent information (provided during recruitment) and were given the option to take another hard copy of the information with them. For participants who had not read the information or wished to be reminded of its contents, the interviewer reviewed key points of the document. Participants were asked if they agreed to be audio-recorded. All said yes without hesitation. Once recording began, each participant was asked again to confirm that she or he agreed to be recorded. At the conclusion of each interview, the interviewer confirmed that she was stopping the recording device. Only individuals able to consent of their own volition were recruited and interviewed.

Processing, Coding, and Analyzing Data

Interview audio files were transcribed in batches by an experienced transcriptionist hired through a local temporary employment agency. Olympus Sonority software was used for audio file playback, with files

removed by KG from the playback software immediately after each transcriptionist's workday concluded. A transcription protocol[1] was used to ensure consistency, and all transcripts were checked against the corresponding audio file by KG, with corrections made as necessary. Completed transcripts were saved in a secure and private folder on the server only accessible to the study team. Transcripts were not returned to participants for review.

KG and KCN used Atlas.ti version 7 to organize and code transcripts. This process was ongoing, as transcripts became available. The interview protocol questions were used to establish a basic coding structure, to which inductive analysis[2] and grounded theory[3] principles were then applied. The inductive analysis process involves open coding to develop codes, categories, patterns, and themes. These elements are then refined, finally using deductive processes to form analytical hypotheses about the data. KG and KCN met regularly (weekly, in most cases) to discuss the coding process and the emerging catalogue of codes. Intercoder reliability was established early in the coding process by way of KG and KCN coding several of the same transcripts and comparing findings, which were overwhelmingly similar. JC and another consultant, Michael Finch, PhD, also met once with KG to review a sample of transcripts and review the accompanying codes that were associated with each interview question. KCN completed the majority of the coding and created a document in which he summarized primary findings by question, in addition to any other notable themes that had emerged.

1. McLellan E, MacQueen KM, Neidig JL. Beyond the qualitative interview: data preparation and transcription. Field Methods. 2003;15(1):63.
2. Patton M. *Qualitative Research and Evaluation Methods.* 3rd ed. ed. Thousand Oaks, CA: Sage; 2002.
3. Glaser B, Strauss, A. . *Discovery of Grounded Theory: Strategies for Qualitative Research.* Chicago: Aldine; 1967.
